# Clinical Characteristics and Diagnostic Correlation of Pediatric Lymphadenopathy in a Secondary-Level Hospital in Colombia

**DOI:** 10.3390/children13040576

**Published:** 2026-04-21

**Authors:** Eyleen Pacheco Narváez, Karina Pastor-Sierra, Nany Castilla Herrera

**Affiliations:** 1 Especialista en Pediatría, Facultad de Ciencias de la Salud, Universidad del Sinú E.B.Z., Montería 230001, Colombia; eylenpacheco@unisinu.edu.co (E.P.N.); nanycastilla@unisinu.edu.co (N.C.H.); 2 Grupo de Investigación Biomédicas y Biología Molecular, Facultad de Ciencias de la Salud, Universidad del Sinú E.B.Z., Montería 230001, Colombia

**Keywords:** lymphadenopathy, lymph nodes, cervical, child

## Abstract

**Highlights:**

**What are the main findings?**

- In this pediatric cohort with lymphadenopathy, cervical involvement predominated and most lymph nodes were ≤1 cm; local inflammatory signs were uncommon.
- Complementary diagnostic testing (biopsy, ultrasound, serology, or tuberculin test) was performed in a small proportion of patients, with no statistically significant differences between rural and urban residence.

**What are the implications of the main findings?**

- Structured clinical assessment and explicit documentation of chart-based assessment features may help standardize initial evaluation and follow-up decisions in pediatric lymphadenopathy.
- Setting-specific data on documentation and complementary test use may inform local protocols for referral, follow-up, and diagnostic work-up in secondary-level pediatric care.

**Abstract:**

Background: Pediatric lymphadenopathy is a common reason for consultation, but information from secondary-level care in Latin American middle-income settings remains limited. Objective: The objective of this study is to describe the clinical profile, documentation quality, and use of complementary tests among pediatric patients with lymphadenopathy treated at a secondary-level hospital in Córdoba, Colombia. Materials and Methods: A retrospective descriptive study was conducted through a review of 246 medical records of children with lymphadenopathy evaluated between January 2020 and June 2024. Sociodemographic variables, lymph node characteristics, local inflammatory signs, associated clinical conditions, and complementary tests were extracted from the charts. An exploratory composite variable of predefined clinical assessment features recorded in the charts was analyzed descriptively. Results: The median age was 6 years, with 58.9% being male, and 79.7% lived in urban areas. Cervical involvement predominated (87.8%); most documented nodes were ≤1 cm (59.3%), and local inflammatory signs were uncommon (7.3%). Complementary testing was limited (complete blood count: 37.8%, serology: 17.9%, tuberculin skin test: 6.9%, ultrasound: 7.7%, and biopsy: 4.1%), without significant rural–urban differences. At least one predefined clinical assessment feature was recorded in 83.7% of charts, most often multiple nodes or bilateral involvement; these findings should be interpreted descriptively rather than as direct indicators of malignancy or infection. Conclusions: In this secondary-level, record-based series, pediatric lymphadenopathy was usually documented as small cervical node enlargement with limited complementary testing. The main contribution of this study is to describe documentation patterns and real-world initial work-up in a Colombian secondary-level hospital, which may inform standardized assessment and referral pathways.

## 1. Introduction

Pediatric lymphadenopathy is a common reason for consultation and is usually related to self-limited infectious or reactive processes. In routine practice, the main clinical challenge is not simply to confirm lymph node enlargement, but to distinguish children who can be managed with clinical follow-up from those who require complementary studies or referral because of persistent, atypical, or progressive findings [1,2,3,4].

Previous reviews and diagnostic algorithms emphasize the value of history taking and physical examination, as well as the selective use of laboratory studies, imaging, and biopsy when warning features are present [4,5,6,7,8]. Additional pediatric reviews and practical approaches, including reports addressing the role of ultrasound in children, support this selective framework [9,10,11,12]. However, much of this evidence comes from referral settings, etiologically focused series, or textbook-level syntheses. Thus, the added value of the present study is not to redefine the classical clinical patterns of pediatric lymphadenopathy, but to document how these patients are recorded and initially worked up in a secondary-level hospital in a Latin American middle-income setting, where local data remain scarce [13,14,15,16].

This perspective is clinically relevant because secondary-level hospitals frequently make the first decisions about observation, testing, and referrals under heterogeneous resource conditions. Even a modest single-center series can therefore provide practice-based information on documentation gaps, the use of complementary tests, and situations in which standardized referral pathways may be needed.

Accordingly, the objective of this study was to describe the clinical characteristics of children with lymphadenopathy treated at a secondary-level hospital in Córdoba, Colombia, with an emphasis on documentation quality, complementary test use, and the distribution of predefined clinical assessment features recorded in the medical charts.

## 2. Materials and Methods

### 2.1. Design of the Study and Study Population

This was a retrospective, descriptive study based on a medical-record review of children and adolescents (0–17 years) with lymphadenopathy evaluated between January 2020 and June 2024 at a secondary-level hospital in Córdoba, Colombia. Because this study was conducted in a single institution, the cohort represents the case mix managed in this care setting and should not be interpreted as population-based.

Sociodemographic variables included age, sex, and area of residence. Residence was classified as urban or rural according to the address category recorded in the medical chart at admission. This variable was analyzed exploratorily as a contextual proxy for potential differences in access to consultation and diagnostic work-up.

When node size or laterality was absent from the chart, the variable was coded as not documented rather than interpreted as a clinical category.

Age was categorized into infant, preschool, school-age, and adolescent groups according to the pediatric age classification used in Colombia. For this reason, age-stratified descriptive analyses were retained, as shown in Supplementary Figure S1.

### 2.2. Statistical Analysis

Data were entered into a Microsoft Excel database and analyzed using R version 4.2.0 (R Foundation for Statistical Computing, Vienna, Austria). Categorical variables were summarized as frequencies and percentages, and age was described using the median and interquartile range. Comparisons between rural and urban groups were exploratory and were performed using the chi-square test or Fisher’s exact test, as appropriate. An exploratory binary logistic regression model was fitted to examine variables associated with predefined clinical assessment features. Given the small number of events in several categories, all inferential analyses were interpreted cautiously.

## 3. Results

### 3.1. Clinical, Sociodemographic, and Paraclinical Characteristics of Pediatric Patients

Table 1 presents the sociodemographic and clinical characterization of the 246 pediatric patients included in this study with a diagnosis of lymphadenopathy, stratified according to their area of residence (rural vs. urban).

The evaluated population consisted of 20.3% of patients from rural areas (n = 50) and 79.7% from urban areas (n = 196). The overall median age was 6 years, with an interquartile range (P25–P75) of 3 to 10 years and minimum and maximum ages of 0 and 17 years, respectively, in both groups. The median age was similar across both settings (6 years in rural patients and 6 years in urban patients), indicating a homogeneous age distribution between the two populations. Regarding sex, a male predominance was observed in both contexts, accounting for 62.0% of rural patients and 58.2% of urban patients and corresponding to 58.9% of the total study population.

In terms of age groups, school-aged children represented the most frequent category both in the overall population (30.5%) and in rural (32.0%) and urban (30.1%) areas. This was followed by the preschool group (28.5% overall; 32.0% rural; 27.6% urban), infants (19.9% overall; 14.0% rural; 21.4% urban), and adolescents (21.1% overall; 22.0% rural; 20.9% urban). These findings suggest a balanced distribution across the different stages of childhood development, with a higher proportion of school-aged children in both environments.

Regarding the clinical characteristics of lymphadenopathy, 51.2% of patients presented with multiple lymph nodes, with virtually identical proportions in rural (50.0%) and urban (51.5%) areas. Laterality was predominantly unilateral (67.5% overall), with higher values in rural settings (72.0%) compared to urban ones (66.3%). Bilateral lymphadenopathy was slightly more frequent in the urban population (31.1% vs. 26.0% in rural areas).

With respect to lymph node size, nodes ≤ 1 cm were the most common (59.3% overall; 56.0% rural; 60.2% urban), followed by those measuring 1–2 cm (19.9% in both settings). Lymphadenopathy > 2 cm was observed exclusively in the urban group (4.1%). In a relevant proportion of charts, lymph node size had not been documented, particularly in rural records (24.0% vs. 15.8% in urban areas); this should be interpreted as incomplete documentation rather than as a clinical subgroup. Regarding the presence of local inflammatory signs, a low overall frequency was observed (7.3%), with a higher proportion in urban areas (8.7%) than in rural areas (2.0%).

### 3.2. Clinical Distribution of Lymphadenopathy by Age Group and Sex

To further characterize the clinical features of patients with lymphadenopathy, three key aspects of the physical examination were explored, namely anatomical location, type of lymphadenopathy (single or multiple), and lymph node size, and analyzed according to age group and sex. Because age groups were defined according to the pediatric classification used in Colombia, the descriptive analysis stratified by age and sex is retained in Supplementary Figure S1.

In Figure S1a, the cervical region was the most prevalent location across all age groups, particularly among school-aged children (67.2%) and preschoolers (59.3%). This pattern also remained when stratified by sex, being more frequent in females (72.0%) than in males (62.8%). Combined lymphadenopathy, defined as the simultaneous presence of lymph nodes in multiple regions, was more frequent in adolescents (23.7%) and in males (31.1%). Inguinal and axillary lymphadenopathies were less common in both analyses.

Figure S1b shows a relatively balanced distribution between single and multiple lymphadenopathies; however, a higher frequency of multiple nodes was observed among preschoolers (64.3%), adolescents (63.9%), and males (54.4%).

Regarding lymph node size (Figure S1c), nodes ≤ 1 cm were the most frequent in all groups, particularly among school-aged children (60.3%) and preschoolers (64.8%). Lymphadenopathy > 2 cm was observed more often in adolescents (5.8%) and in male patients (5.6%). A considerable proportion of records lacked size specification (up to 18.2% in preschoolers and 18.8% in the female group), which limits objective assessment.

### 3.3. Characterizing the Diagnostic Approach in Pediatric Patients with Lymphadenopathy

To improve readability, Table 2 only summarizes the frequency of complementary tests actually performed, both overall and according to area of residence. This comparison was exploratory and intended to characterize the diagnostic work-up used in the study setting.

Regarding etiological confirmation procedures, lymph node biopsy was performed in only 4.1% of the total patients, with a slightly lower proportion in rural areas (2.0%) than in urban areas (4.6%) and no statistically significant differences (*p* = 0.6919). Thus, histological confirmation was only available for a small subset of cases.

With respect to serological testing, the TORCH/HIV panel was requested in 17.9% of patients, with similar frequencies in rural (20.0%) and urban areas (17.3%) (*p* = 0.8179). Among tested patients, acute serological positivity was uncommon: 2.4% for Toxoplasma gondii, 2.4% for cytomegalovirus (CMV), and 2.0% for Epstein–Barr virus (EBV).

The tuberculin skin test, which is used to screen for tuberculous lymphadenitis, was performed in 6.9% of the pediatric population, with similar proportions in rural (8.0%) and urban areas (6.6%) (*p* = 0.7558).

Regarding diagnostic imaging, ultrasound was performed in 7.7% of patients, with a higher proportion in rural areas (16.0%) than in urban areas (5.6%), although without statistically significant differences (*p* = 0.2049).

Concerning basic laboratory tests, a complete blood count (CBC) was reported in 37.8% of patients, with similar frequencies in rural (38.0%) and urban areas (37.8%) (*p* = 0.9897). In addition, among acute-phase reactants, C-reactive protein (CRP) and the erythrocyte sedimentation rate (ESR) were requested in approximately half of the patients, whereas lactate dehydrogenase (LDH) was assessed in a smaller proportion, with no statistically relevant differences between rural and urban settings.

Overall, complementary tests were documented in a minority of records, and no statistically significant differences according to residence were observed. These findings should be interpreted primarily as a description of documentation and diagnostic practice rather than as evidence of differential etiologic investigation between groups.

### 3.4. Identification of Predefined Clinical Assessment Features and Construction of an Exploratory Composite Variable

For exploratory purposes, a composite variable termed “predefined clinical assessment features” was constructed from chart-documented findings commonly cited in pediatric reviews and diagnostic algorithms for lymphadenopathy evaluation [4,5,6,7,8,17]. These variables were used as chart-based descriptors that may prompt closer clinical assessment or follow-up; they were not intended to classify malignancy or infection. The variable was coded dichotomously (yes/no) when at least one of the following was present:

- Lymph node size > 2 cm
- Hard lymph node consistency
- Symptom duration > 6 weeks
- Bilateral involvement
- Presence of local inflammatory signs
- Multiple-lymph node involvement.

This composite measure was only used for descriptive and exploratory analytical purposes. It was not designed or validated to predict malignancy or infection, and the inclusion of bilateral involvement or multiple nodes should be interpreted as contextual chart-based features rather than as isolated markers of severe disease.

**Figure 1:**
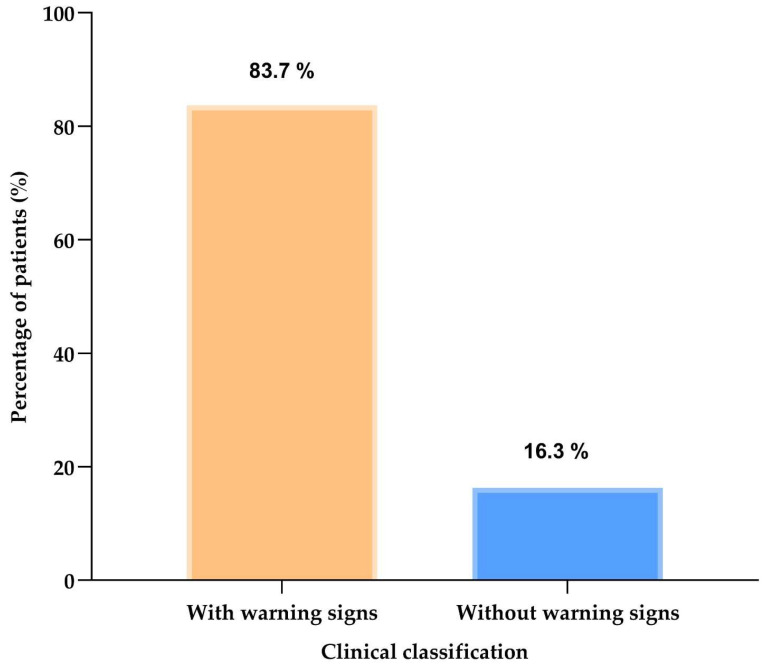
Overall proportion of pediatric patients with at least one predefined clinical assessment feature recorded in the chart (n = 246).

Cohort analysis showed that 83.7% (n = 206) of patients had at least one predefined clinical assessment feature, whereas 16.3% (n = 40) had none. Figure 1 presents this overall distribution.

**Figure 2:**
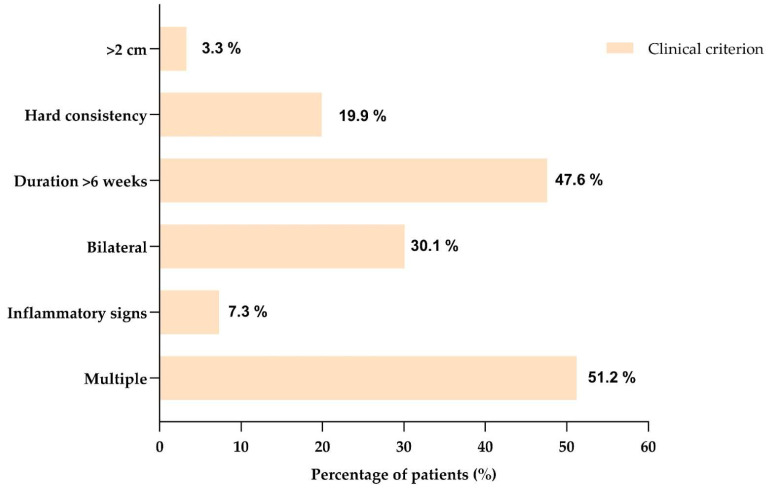
Individual frequency of the predefined clinical assessment features included in the exploratory composite variable (n = 246).

When each component of the composite variable was examined separately (Figure 2), the most frequent findings were multiple lymphadenopathy (51.2%) and bilateral involvement (29.7%), followed by symptom duration >6 weeks (22.4%) and local inflammatory signs (7.3%). Lymphadenopathy > 2 cm and hard consistency were less frequent (4.1% and 3.7%, respectively).

### 3.5. Association Between Clinical Characteristics and the Presence of Predefined Clinical Assessment Features

**Figure 3:**
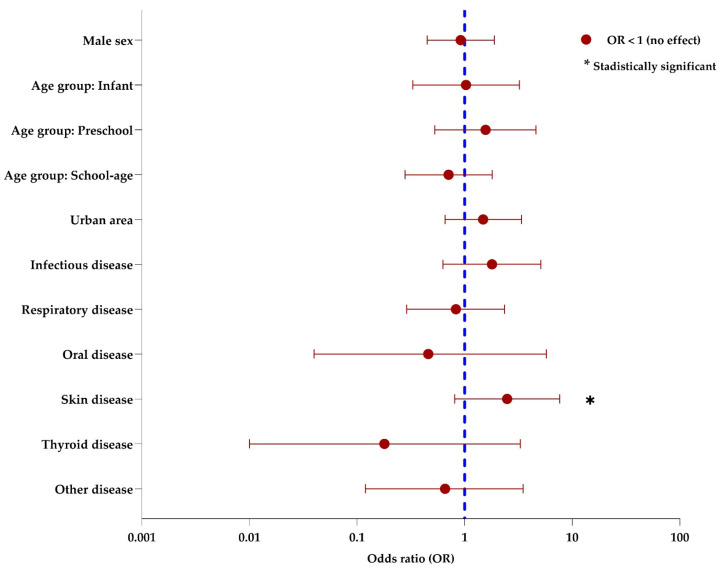
Exploratory multivariable analysis of factors associated with predefined clinical assessment features in pediatric lymphadenopathy. The dashed vertical line marks OR = 1; confidence intervals crossing 1 indicate no clear association.

To explore factors associated with the presence of predefined clinical assessment features, an exploratory binary logistic regression model was fitted (Figure 3). Table S1 presents the odds ratios (ORs), 95% confidence intervals (95% CIs), and *p*-values for each variable.

The model included sociodemographic variables (sex, age group, and area of residence) and grouped clinical variables derived from the medical record (associated skin, respiratory, infectious, thyroid, oral, or other pathology; acute-phase reactants; and complete blood count results). Because several categories had low frequencies, the model estimates should be interpreted as imprecise.

None of the sociodemographic variables showed a statistically robust association with predefined clinical assessment features. Effect estimates across age groups were heterogeneous, but confidence intervals were wide. Area of residence was not significantly associated with predefined clinical assessment features (OR: 1.49; *p* = 0.340).

Among the grouped clinical variables, associated skin pathology showed the largest positive point estimate (OR: 2.49; 95% CI: 0.81–7.65), but the confidence interval was wide and crossed the null value. Therefore, this finding should be interpreted cautiously and not as conclusive evidence of association.

The remaining grouped clinical variables, including respiratory, infectious, thyroid, and oral pathologies, did not show clear associations with predefined clinical assessment features in this exploratory model, and all corresponding estimates were imprecise. Overall, the results of the multivariable analysis should be interpreted as hypothesis-generating rather than confirmatory.

## 4. Discussion

This study describes how pediatric lymphadenopathy is documented and initially investigated in everyday practice at a secondary-level hospital in Córdoba, Colombia. The dominant pattern was small cervical nodes with few local inflammatory signs, while complementary testing and etiologic confirmation were uncommon. Therefore, the main contribution is not the proposal of a novel clinical phenotype, but the description of documentation practices and first-line diagnostic behavior in an underreported care setting.

The value of these findings lies less in defining the exact causes of lymphadenopathy and more in showing how children with this condition present at first contact and how they are assessed in routine care. This perspective is relevant in secondary-level services, where the initial decision is often whether observation is sufficient or whether laboratory tests, imaging, or referral are needed. 

One aspect that deserves attention is the limited use of complementary tests in the reviewed records. This should not be interpreted automatically as inadequate evaluation because a retrospective chart review does not capture the full clinical reasoning behind each decision. Rather, it suggests heterogeneous diagnostic practices that may reflect both the predominantly mild clinical profile of many cases and the absence of standardized local pathways for documentation and follow-up. In this context, ultrasound can be considered an adjunctive tool for morphologic assessment, but its interpretation should remain integrated with the clinical picture and follow-up needs [18,19].

A similar caution is needed when interpreting the exploratory composite variable of predefined clinical assessment features. In this series, that variable was frequently driven by the presence of multiple nodes or bilateral involvement, findings that are relatively common in pediatric practice and that, by themselves, do not indicate severe disease. For that reason, the coexistence of these features and a modest rate of diagnostic testing should be read carefully and not used as direct evidence of malignancy, infection severity, or underassessment.

Our results are broadly in line with pediatric reports showing that cervical lymphadenopathy is the most common presentation and that many children can initially be managed on the basis of history and physical examination [1,4,5,12,17,20]. However, the present study contributes local evidence from a Colombian secondary-care setting, where patterns of access, referral, and documentation may differ from those described in tertiary centers or high-income countries.

By contrast, our data reflect the first stages of evaluation in routine pediatric care, which helps explain why most recorded cases had a low-complexity profile and why confirmatory testing was not common.

This study also exposed important limitations in the clinical record itself. Variables such as symptom duration, node consistency, the precise indication for complementary tests, and follow-up plans were not documented uniformly. These gaps reduced the interpretability of some findings and made it difficult to compare our results with those of studies based on prospectively collected data.

Several limitations should therefore be acknowledged. This study was retrospective, relied on routine medical records, and was conducted at a single institution. The cohort is not population-based, and children referred directly to tertiary centers may not be represented. In addition, the low frequency of imaging and biopsy prevents any firm conclusion regarding malignant or other less common etiologies. Likewise, histopathological details of the few biopsied cases, as well as information on prior treatment with antibiotics or anti-inflammatory drugs before referral to the secondary-level hospital, were not consistently documented in the medical records and therefore could not be analyzed reliably.

Even with these limitations, the data support a practical message relevant to daily care: careful history taking and physical examination remain the foundation of the initial assessment of pediatric lymphadenopathy. Better local guidance on when to request complementary tests and when to refer patients could improve consistency without encouraging indiscriminate testing.

Future prospective studies with standardized definitions, follow-up, and explicit diagnostic criteria would help clarify the role of laboratory tests, ultrasound, and biopsy in secondary-level pediatric care. They would also allow a more reliable description of etiologic distribution, including uncommon but clinically important causes [21,22,23].

## 5. Clinical Recommendations

1. Detailed clinical evaluation: Physicians should prioritize careful history taking and physical examination to characterize location, size, duration, consistency, and associated signs.
2. Selective use of diagnostic tests: Physicians should request laboratory studies, imaging, or biopsy according to the clinical scenario, persistence, and concerning features, as well as establish explicit criteria for reassessment and escalation when concerning chart-based features persist, increase, or cluster during follow-up.
3. Standardized documentation: Consistent recording of node size, consistency, duration, inflammatory signs, and follow-up plan in the medical record should be promoted.
4. Management protocols: Management should develop and implement local clinical guidelines that establish clear criteria for evaluation, follow-up, and referral of children with lymphadenopathy.
5. Appropriate follow-up: Physicians should ensure clinical reassessment of persistent or atypical cases and refer them to specialists when evolution or associated findings warrant broader evaluation.

## 6. Conclusions

In this retrospective series from a secondary-level hospital in Córdoba, Colombia, most children with lymphadenopathy presented with cervical involvement, lymph nodes ≤ 1 cm, and a low frequency of local inflammatory signs. These findings describe the clinical profile documented at initial evaluation and should be interpreted mainly as evidence of routine presentation and documentation patterns in this care setting.

Complementary diagnostic tests were performed in a minority of cases, and no significant differences were observed according to residence. Because etiologic confirmation was limited, this study should not be interpreted as establishing the prevalence of infectious or malignant causes. Rather, it provides local descriptive evidence that may support the development of more standardized assessment, documentation, and referral pathways for pediatric lymphadenopathy.

## Figures and Tables

**Table 1:** Sociodemographic and clinical characteristics of pediatric patients with lymphadenopathy according to their area of residence in the Department of Córdoba.

Variable	Total (n = 246)	Rural (n = 50)	Urban (n = 196)
Age (Median [P25–P75], [min–max])	6.0 (3.0–10.0) [0–17]	6.0 (3.0–9.0) [1–17]	6.0 (3.0–10.25) [0–17]
Sex n (%)			
Female	101 (41.1)	19 (38.0)	82 (41.8)
Male	145 (58.9)	31 (62.0)	114 (58.2)
Age group n (%)			
Infant	49 (19.9)	7 (14.0)	42 (21.4)
Preschool	70 (28.5)	16 (32.0)	54 (27.6)
School-age	75 (30.5)	16 (32.0)	59 (30.1)
Adolescent	52 (21.1)	11 (22.0)	41 (20.9)
Type of lymphadenopathy n (%)			
Single	120 (48.8)	25 (50.0)	95 (48.5)
Multiple	126 (51.2)	25 (50.0)	101 (51.5)
Laterality n (%)			
Unilateral	166 (67.5)	36 (72.0)	130 (66.3)
Bilateral	74 (30.1)	13 (26.0)	61 (31.1)
Not documented	6 (2.4)	1 (2.0)	5 (2.6)
Lymph node size n (%)			
Not documented	43 (17.5)	12 (24.0)	31 (15.8)
≤1 cm	146 (59.3)	28 (56.0)	118 (60.2)
1–2 cm	49 (19.9)	10 (20.0)	39 (19.9)
>2 cm	8 (3.3)	-	8 (4.1)
Inflammatory signs n (%)			
No	228 (92.7)	49 (98.0)	179 (91.3)
Yes	18 (7.3)	1 (2.0)	17 (8.7)
Lymphadenopathy region n (%)			
Cervical	216 (87.8)	43 (86.0)	173 (88.3)
Inguinal	12 (4.9)	4 (8.0)	8 (4.1)
Axillary	8 (3.3)	1 (2.0)	7 (3.6)
Combined regions	10 (4.1)	2 (4.0)	8 (4.1)

**Table 2:** Frequency of complementary tests performed in pediatric lymphadenopathy cases according to area of residence.

Diagnostic Test	Total n (%) (n = 246)	Rural n (%) (n = 50)	Urban n (%) (n = 196)	*p*-Value
Lymph node biopsy	10 (4.1)	1 (2.0)	9 (4.6)	0.6919
Serology (Toxoplasma/CMV/EBV/HIV)	44 (17.9)	10 (20.0)	34 (17.3)	0.8179
Tuberculin skin test	17 (6.9)	4 (8.0)	13 (6.6)	0.7558
Ultrasound	19 (7.7)	8 (16.0)	11 (5.6)	0.2049
Complete blood count (CBC)	93 (37.8)	19 (38.0)	74 (37.8)	0.9897

## Data Availability

The data may be available from the corresponding author upon reasonable request.

## Supplementary Materials

The following supporting information can be downloaded at https://www.mdpi.com/article/10.3390/children13040576/s1, Table S1. Multivariable logistic regression analysis of factors associated with the presence of predefined clinical assessment features in pediatric lymphadenopathy. Figure S1. Age- and sex-stratified distribution of the anatomical location, type of lymphadenopathy, and lymph node size: (a) anatomical location, (b) type of lymphadenopathy, and (c) lymph node size.

## Abbreviations

The following abbreviations are used in this manuscript:

CBC	complete blood count
CI	confidence interval
CMV	cytomegalovirus
CRP	C-reactive protein
EBV	Epstein–Barr virus
ESR	erythrocyte sedimentation rate
HIV	human immunodeficiency virus
LDH	lactate dehydrogenase
OR	odds ratio
TORCH	toxoplasmosis, other agents, rubella, cytomegalovirus, and herpes simplex
