# Supplementary material for: Clinical Characteristics and Diagnostic Correlation of Pediatric Lymphadenopathy in a Secondary-Level Hospital in Colombia
Source: Children (Basel). 2026 Apr 21;13(4):576. doi: 10.3390/children13040576 (PMC13114521; doi:10.3390/children13040576)
Supplement: Supplementary file 1 [file children-13-00576-s001.zip › children-4142386-supplementary.pdf]

children

MDPI

a.

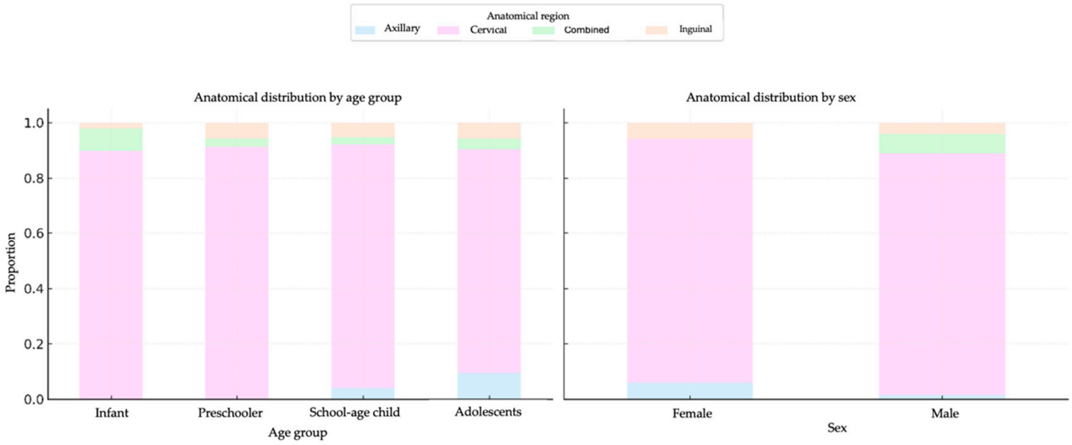

b.

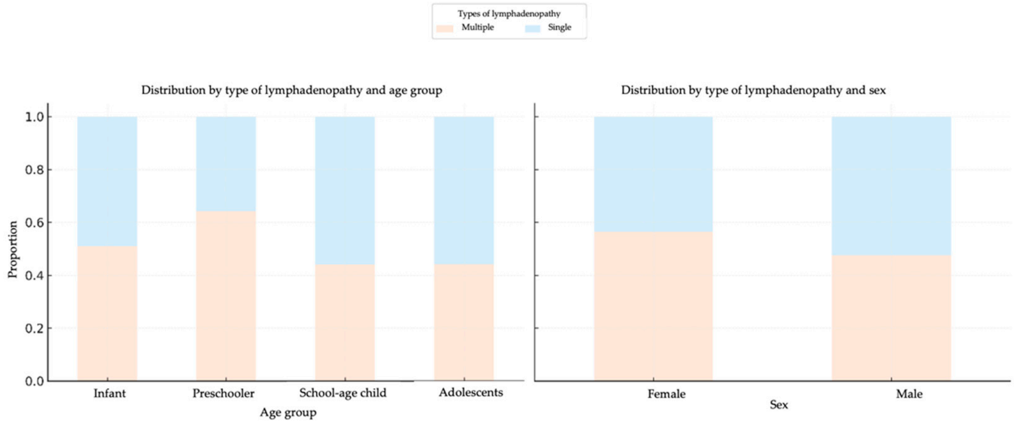

c.

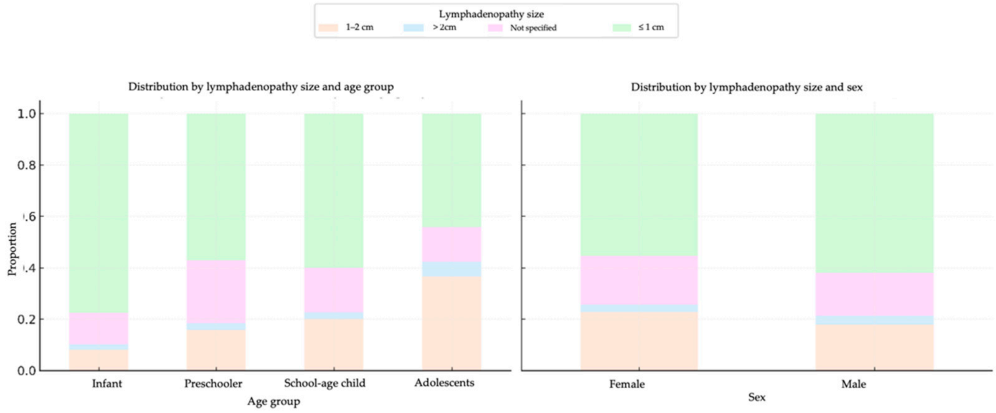

Figure S1. Age- and sex-stratified distribution of (a) anatomical location, (b) single versus multiple lymphadenopathy, and (c) lymph node size.

children

MDPI

Table S1. Multivariable logistic regression analysis of factors associated with the presence of predefined clinical assessment features in pediatric lymphadenopathy.

Independent variable	OR	95% CI (Lower–Upper)	p-value
Intercept	3.54	1.19–10.49	0.023
Male sex	0.92	0.45–1.89	0.817
Age group: Infant	1.03	0.33–3.23	0.955
Age group: Preschool	1.57	0.53–4.61	0.412
Age group: School-age	0.71	0.28–1.81	0.470
Urban area of residence	1.49	0.66–3.39	0.340
Infectious comorbidity	1.80	0.63–5.12	0.999
Respiratory comorbidity	0.83	0.29–2.35	0.726
Oral comorbidity	0.46	0.04–5.74	0.548
Skin comorbidity	2.49	0.81–7.65	0.048
Thyroid comorbidity	0.18	0.01–3.29	0.250
Other comorbidity	0.66	0.12–3.51	0.625

Note: OR, odds ratio; CI, confidence interval. Estimates should be interpreted cautiously because several categories had low frequencies.
